# Global Comparisons of Age, Gender and Socioeconomic Status Differences of Physical Fitness Health Risk in South African Primary School Children: Longitudinal Data from the NW-CHILD Study

**DOI:** 10.3390/ijerph21121554

**Published:** 2024-11-25

**Authors:** Xonné Muller, Anita E. Pienaar, Barry Gerber, Colin N. Moran, Naomi E. Brooks

**Affiliations:** 1 Physical Activity, Sport and Recreation Focus Area, Faculty of Health Science, North-West University, Potchefstroom 2520, South Africa; anita.pienaar@nwu.ac.za (A.E.P.); barry.gerber@nwu.ac.za (B.G.); 2 Department of Human Movement Sciences, University of Fort Hare, Alice 5700, South Africa; 3 Faculty of Health Sciences and Sport, University of Stirling, Scotland FK9 4LA, UK; colin.moran@stir.ac.uk (C.N.M.); n.e.brooks@stir.ac.uk (N.E.B.); 4 Department of Kinesiology, DePauw University, Greencastle, IN 46135-0037, USA

**Keywords:** physical fitness, health risk factors, muscular strength, primary school children, aerobic capacity, cardiorespiratory fitness, muscular fitness, criterion reference standards

## Abstract

Global physical fitness (PF) levels have declined over the past 50 years, contributing to early health risks in children although it is still unclear how age, gender and socioeconomic status (SES) impact PF risk. This study aimed to identify unique health risks related to age, SES and gender that might influence muscular fitness (MF) and cardiorespiratory fitness (CRF) levels of primary school children in South Africa. Children (*N* = 349, boys = 165; girls = 184) of low (*n* = 201) and high SES (*n* = 148) underwent three time point measurements at 6, 9 and 12 years spanning seven primary school years. MF was assessed with the strength sub-test of the Bruininks–Oseretsky Test of Motor Proficiency (BOT-2) and CRF with a 20 m shuttle run. Relationships between biographical data, anthropometric data and PF were analysed using mixed linear regression models. After controlling for covariates, findings from unadjusted interaction models were used, revealing significant (*p* < 0.05) two-way age and SES interactions in standing long jump (SLJ), push-ups, wall-sit, sit-ups and VO_2_max and age and gender interactions (*p* < 0.001) in sit-ups, push-ups and VO_2_max. Universal cut-points are considered valid monitoring guidelines of PF risk in South African school children. For comparison, standardised global protocols for sit-ups and push-ups should be prioritised and intervention strategies should focus on improving PF in girls, older children from the age of 9 and children from low SES backgrounds.

## 1. Introduction

The global decline in the physical fitness (PF) and physical activity (PA) levels of children over the past decades emphasises the importance of monitoring secular trends [1,2]. Shifts in PF and PA levels across generations serve as valuable indicators of health risks and should be employed for future health-monitoring purposes [3]. Health-related PF includes components that are used to monitor health, such as aerobic capacity, also known as cardiorespiratory fitness (CRF), muscle strength and endurance, flexibility and body composition [4]. Several factors, including changes in means of transportation, access to recreational activities and safe environments, reduced PE in schools, technology, schoolwork, including studying for tests, and increased sedentary behaviour, are associated with declining PA and PF levels [3,5]. These increased sedentary trends negatively impact physical health and also adversely affect global health systems and economic growth [6].

CRF is one of the vital health risk indicators for cardiovascular health during childhood and adolescence [7]. Lower body strength assessed through standing long jump (SLJ) in youth is also a health marker that can act as a practical and economical strategy to assess health in children from low- to middle-income countries (LMICs), such as South Africa. Because muscular strength has also been associated with vigorous PA and health risks, more data to clarify the importance of this relationship are vital, especially for LMICs [8,9].

Gender is one of the main factors that influence PF in apparently healthy children between the ages of 6 and 12 years [10]. Boys are commonly described as being more physically active than girls, contributing to higher PF levels, specifically in strength and aerobic capacity tests [10,11,12,13,14,15]. Several researchers have already established gender and age-specific cut-points for CRF to assist with early identification of health risks in children and adolescents aged 7–19 years old [5,7,16,17]. The Fitnessgram is a leading global toolkit for assessment of health-related fitness in children and adolescents aged five and above that provides gender- and age-specific cut-points to classify youth as in, above or below the healthy fitness zone (HFZ) [18]. Age- and gender-specific CRF norms for European children were updated by Tomkinson et al. [17] as these norms, established 10 years ago, were outdated. Ruiz et al. [7] identified gender-specific cut-points for the assessment of CRF to assist in identifying cardiovascular health risks in children aged 6 to 19 years old which are also recommended to be universally utilised for comparative reasoning [5].

PF is currently used as a primary health marker to determine the prevalence of non-communicable diseases (NCDs), to combat overweight and obesity as the main mode of intervention and in prevention strategies to increase PF and PA globally. Great disparities in CRF levels are also revealed between northern and southern countries [16]. In this regard, the Global Action Plan on Physical Activity emphasises the identification of risk factors that can counter PA participation which can lead to disparities between populations [6]. It is further imperative to establish a standard instrument of PA and PF measurements for global comparability [19]. Hence, PF remains a risk factor in question and needs more understanding from the scientific community [12,20] since age, gender and SES can also be influential factors [3,11,21,22]. Obesity and undernutrition, which are global health concerns, are also prevalent in the South African child population and are further associated with poor PF levels [10,11,12,13].

Urbanisation in developing countries such as South Africa (S.A) could have a negative impact on PF levels, which are of national health concern [23,24,25]. Researchers should also focus on investigating the impact of SES on PF levels and, consequently, health risks among South African children [15,25,26,27,28]. National assessments of PF should be performed annually across the globe, including in South African schools [23]. Additionally, research regarding the development of toolkits related to fitness measurements is recommended to aid in evaluating these health risks [22]. Universal or national cut-points based on age and gender will assist in comparing healthy fitness levels across all provinces in S.A. These recommendations include the implementation of a national assessment plan to track PF levels in schools. This recommendation provides a foundational framework for understanding the reportedly low PF results in lower-SES groups and offers valuable insights into environmental effects for both researchers and practitioners [5,15]. Researchers found that physical fitness (PF) levels among South African children are influenced by SES in various provinces of S.A, with low-SES groups being associated with lower PF levels [26,29]. Younger children (8–9 years) have higher CRF scores than older children (10–15 years) [15], indicating that they are healthier [5,30].

The occurrence of poor performance in PF tests, as well as a lack of specific cut-points and norms in lower-SES environments within S.A, urges the importance of investigating the relevancy of using international fitness standards for comparative reasoning [15,31]. In addition, the necessity to further investigate the influence of age, SES and body composition on PF test performance over a longitudinal period also arises [10,32]. Therefore, practice-base evidence of PF as part of active monitorisation to assist in needs analysis for identifying interventions is crucial as it seems that younger children exhibit higher levels of PF [6]. Even though several studies report on factors related to PF of South African children, those had small samples, mostly included adolescents, were limited to one province of S.A, were cross-sectional or lacked gender or SES disparities [26,27,33,34,35]. Tracking PF trajectories over the long term can assist in health promotion and intervention through the development of toolkits [6,23].

This study aims to determine longitudinal changes in PF levels of South African primary school children, considering age, gender, body composition and SES influences and comparing the results with universal standards and global studies. Such findings can be used to monitor high-risk and inactive fitness behaviour in children to inform policymakers and to emphasise the need for interventions and health risk prevention. Should a need arise from these analyses, unique PF thresholds can also be established for the South African socioeconomic context via the longitudinal evidence from this population of 6- to 13-year-old children in the NW province of S.A.

## 2. Materials and Methods

### 2.1. Research Design

This research is a sub-study of the Child Health Integrated Learning and Development (NW-CHILD) longitudinal study that was conducted in 1 of the 9 provinces of South Africa from 2010–2016, with three time points (2010, 2013 and 2016) over the primary school years, including 20 randomly selected schools. Around 8% of the S.A population lives in North West Province (NWP) which is marked by extreme levels of unemployment and poverty (59.6%), particularly in rural communities (statistics SA, 2019). These statistics emphasise the poor circumstances in the NWP compared to the 32.9% national unemployment and 40% poverty rates [36,37]. Each district includes schools from quintiles one to five, with schools in quintiles one to three having low SES and those in quintiles four and five representing high-SES schools according to a classification system used in S.A [32]. The sampling procedures for the NW-CHILD study are thoroughly explained in detail elsewhere by de Waal and Pienaar [38]. Children had to remain in the same school to be included in the study, thus the same SES classification was applicable over the seven-year timespan.

### 2.2. Ethical Considerations

The NW-CHILD study obtained ethical clearance from North-West University’s (NWU) ethical committee (00070-09-A1). The North West Department of Basic Education (DBE) also approved the study. Ethical clearance was further obtained for this sub-study (00070-09-A1-04). Consent was also received from the participating schools’ principals for performing tests during school time at the schools. Furthermore, every parent also received an informed consent form to complete. Children whose parents provided consent for their participation also had to give assent when younger than 8 years or consent when older for participation in the study. Consent was obtained at each of the time point measurements.

### 2.3. Study Population and Procedures

**Figure 1:**
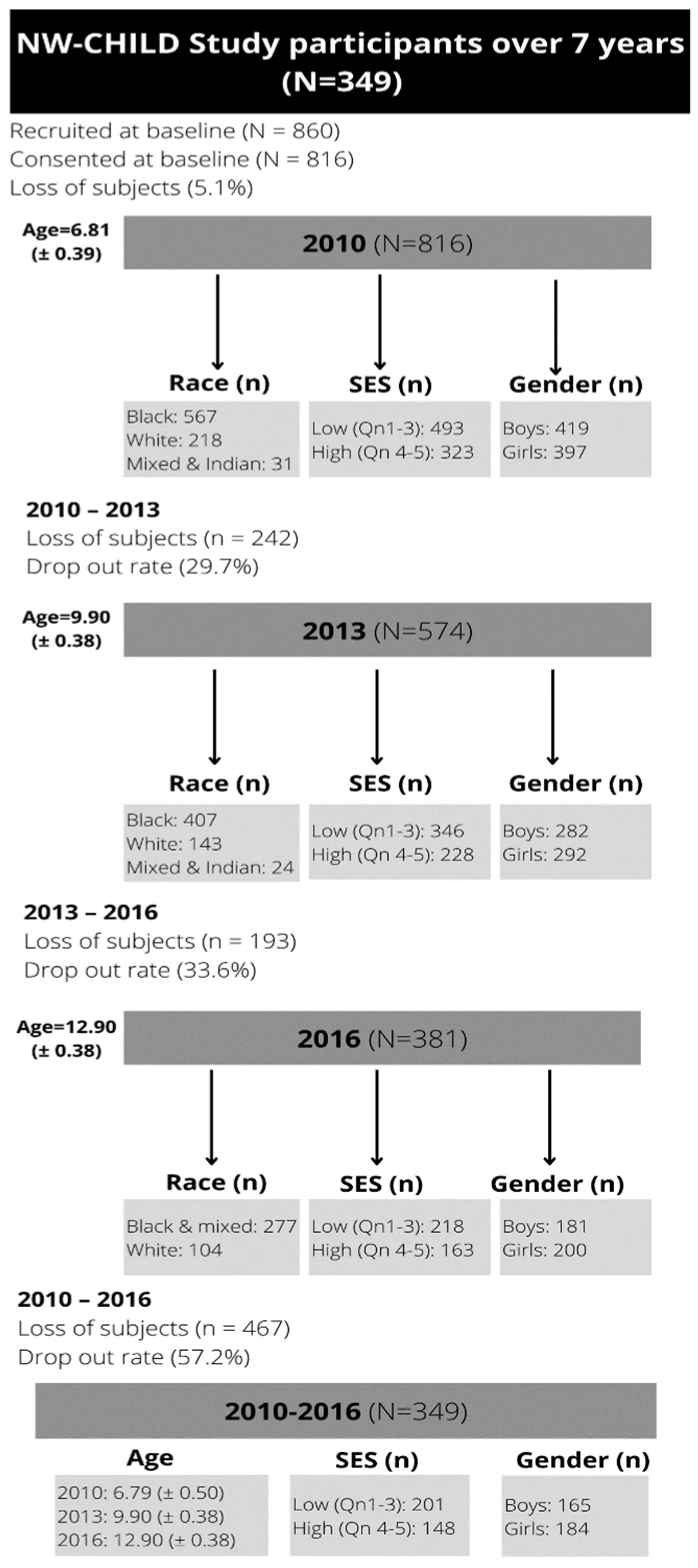
Participant recruitment for the NW-CHILD study (2010–2016).

Figure 1 provides a breakdown of the total participants and classification according to gender and SES. The mean age and distribution of participants at each time point measurement are also indicated in Figure 1. The initially recruited 860 participants who were enrolled from 20 primary schools in 2010 (Grade 1) at baseline were 6.8 years (±0.39) of age. Figure 1 provides an overview of the participant sample, attrition rate and mean age at each time point measurement. Although 829 parents consented for children to participate, on the day of testing only 816 participated (419 boys and 397 girls; 5.1% dropout ratet) because of absence on the testing day, relocation and moving to another school or exclusion because of inaccurate ages. For the first follow-up measurement, 574 participants (282 boys and 292 girls; dropout rate 29.7%) aged approximately 9 years (±0.39) consented to the study. At the final time point measurement in 2016, 381 participants (181 boys and 200 girls) consented, indicating a further drop out of 33.6%. The study experienced a total dropout rate of 53.31% (n = 435) over the 7-year follow-up period. High dropout rates are not uncommon in South African schools due to rural-–urban migration in S.A, especially over the 7-year school period [39].

This study focused on all participants who took part in all three measurements in 2010, 2013 and 2016 to be included in this study, except for the PACER tests since aerobic capacity is not recommended to be tested for children younger than 10 years old. All children that presented with physical disabilities and whose parents did not consent were excluded from the study. The layout and set-up of stations varied between school settings according to available space, with the CRF test (PACER) performed after all other testing was concluded. All testing was conducted by postgraduate students, trained beforehand in all the testing procedures, and senior researchers with specialisation in paediatric movement science. Translators were available when English or Afrikaans was not the participant’s first language.

### 2.4. Measurement Instruments and Apparatus

#### 2.4.1. Anthropometric Measurements

Anthropometry, which included stature (cm), body mass (kg), measurement of the sub-scapular, calf and triceps skinfolds (mm), body fat percentage (%) relaxed forearm and waist circumferences (cm), were measured before any fitness testing commenced. All procedures were performed according to the International Society for the Advancement of Kinanthropometry (ISAK) protocols [40]. A Harpenden portable stadiometer (Holtain Ltd., Crymych, UK) and an electronic scale (BF 511, Omron; Omron healthcare Co. Ltd., Kyoto, Japan) were used to measure stature and body weight to the nearest 0.1 cm and 0.1 kg. BMI was calculated by using stature and mass (body mass in kg divided by height in m^2^). The BMI classification was used to determine thinness, overweight or normal weight in gender- and age-specific categories according to Cole et al. [41]. Additionally, the BF 511 Omron was also used to measure body composition in terms of fat-free mass and body fat. Circumferences were taken twice to ensure validity and reliability and were measured with a metal measuring tape (Cescorf, Porto Alegre, Brazil). Skinfolds were also taken twice with a Harpenden skinfold calliper to provide an average value. This takes into consideration the technical error of measurement. Height-for-age (HAZ)-2 z-scores calculated with Anthroplus [42] were used to classify children as stunted. Firstly, two researchers measured all anthropometry, whereafter the fitness testing took place with the PACER test as the final measurement of the day.

#### 2.4.2. Progressive Aerobic Cardiovascular Endurance (PACER) Test

The PACER test is part of the Fitnessgram testing protocol for assessment of aerobic fitness. It is a multi-level 20 m progressive running test that assesses aerobic capacity through estimating VO_2_max (milliliters of O_2_ consumed per kilogram of body weight per minute, or mL/kg/min) [18]. Participants are required to run back and forth between 20 m lines as many times as they can on the cue of a “beep” sound while the test gets progressively faster each minute. The test continues until the participant fails to reach the 20 m line for the second time. The maximum number of laps is then recorded to determine the VO_2_max which is predicted by using the number of PACER laps and the age of the participant in the following equation: VO_2_max = 44.862 + (PACER × 0.347) − (Age × 1.050) [18]. The score is used to classify participants into various healthy fitness zones (HFZs), i.e., 2 = in or above the HFZ; 1 = the needs improvement zone and 0 = the needs improvement—health risk zone [18,43]. The PACER test was only administered in Grades 4 and 7 (9 and 12 years old) in the study, as no reference standards are available for children younger than 10 years due to the apprehension of younger children pacing themselves accurately and to ensure that they produce a maximal effort [4]. Apart from the PACER test that was outside on the sports field, most other tests took place in a hall, classroom or sick bay to ensure privacy.

#### 2.4.3. Bruininks–Oseretsky Test of Motor Proficiency, Second Edition (BOT-2)

This test battery was individually administered through assessing motor skills via goal-directed activities that can subsequently be used to assess motor proficiency. Essentially, the BOT-2 assessed various motor proficiency areas in children aged 4 to 21 years old and uses norm-referenced criteria with confirmed validity and reliability (r = 0.80–0.95). Assessment takes approximately 20–30 min to conclude, with an extra 10 min to prepare the test area [44]. The criteria for the motor areas are structured according to muscle groups and extremities and include four different test categories, i.e., fine manual control, manual coordination, body coordination and strength and agility. Only the short-form version (BOT-2 SF), which included 14 items, was used in the NW-CHILD study. The complete set of test items of the complete form for strength (standing long jump; wall-sit; push-ups; sit-ups; and the v-up), were, however, assessed additionally and all these test items were specifically used as additional indicators of fitness for this study. Participants were allowed two trials, and the best score was used as the raw score for each item to convert into point scores via the conversion tables provided in the administering booklet. The sum of point scores for strength sub-tests was calculated to obtain the scale score (SS), using gender-specific conversion tables (B1 and B2) in the BOT-2 manual for interpreting fitness performance and descriptive categories. The descriptive categories were applied as follows: well above average ≥ SS 25; above average = 20–24; average = 11–19; below average = 6–10; well below average ≤ 5 [44].

### 2.5. Statistical Analysis

The Statistical Package for the Social Sciences (SPSS) for Windows, version 27 [45] (IBM Corp, Armonk, New York, 2020) was used to calculate descriptive statistics for all variables in the study. Categorical variables are reported as frequencies and percentages. Means and standard deviations or medians and interquartile ranges are reported for continuous variables, depending on the distribution of the variables. A previous study by Pienaar [27] reported an analysis of the same sample population indicating that no bias was caused by the loss of subjects over different time points. The data were assumed to be missing at random and did not significantly affect the results.

A linear mixed model with repeated measures was used to investigate the relationships between biographical data (age, gender and SES), anthropometric data (height, weight, body fat, skinfolds, z-scores, BMI, thinness and stunting) and physical fitness tests (muscular strength and endurance and aerobic endurance). The time variable (2010–2016) was treated as a fixed effect and physical fitness as the response variable. Plots of standardised residuals were used to evaluate the assumptions of normality, homoscedasticity, linearity, independence of errors and the absence of outliers. An adjusted Cohen’s d-value (Tukey post hoc test) was calculated to determine practical significant differences between standardised means over time (2010–2016). The following guideline values were used to classify an effect size: small (d = 0.2), denoting a practically non-significant difference, medium (d = 0.5), indicating visible practical difference, and large (d = 0.8), implying a practical significant difference [46]. Interaction models were fitted for BMI, body fat percentage (BFP), stature and weight. To control for influences of malnutrition, separate models were also fitted to determine if dependent variables (strength tests) significantly differ between thinness and stunted groups compared to normal-weight children. No significant differences were found in the adjusted models except on three occasions, however, the stunted group was relatively small (*n* = 6). Based on these findings from the additional adjusted models no further distinction of specific models was found necessary between normal-weight and malnourished groups. Thus, the unadjusted models were used to report results.

## 3. Results

This study included 349 participants, 165 boys and 184 girls of high (boys = 82; girls = 66) and low (boys = 83; girls = 118) SES, which were tested over 3 time periods, i.e., 2010, 2013 and 2016. With regard to SES, quintile 1–3 schools represented 58% (*n* = 201) and quintile 4–5 schools 42% (*n* = 148) of the sample. These participants had a mean age of 6.89 ± 0.50 in 2010 (Grade 1), 9.90 ± 0.38 in 2013 (Grade 4) and 12.90 ± 0.38 in 2016 (Grade 7). For discussion purposes, the study will refer to the average ages of 6, 9 and 12 years old.

The descriptive characteristics for age, anthropometric (body mass, stature, body fat percentage, skinfolds) and fitness measurements (CRF, muscular strength and muscular endurance) of the group (*N* = 349) for each time point measurement, according to SES and gender, are presented in Table 1. Gender differences in stature and body mass are notable where children of a high SES were consistently taller (boys = approximately 8 cm and girls = approximately 5 cm) and heavier (boys ≥ 5 kg and girls ≥ 4 kg) at all time point measurements compared to those of a low SES. These differences were also observed in all the other anthropometric measurements.

**Figure 2:**
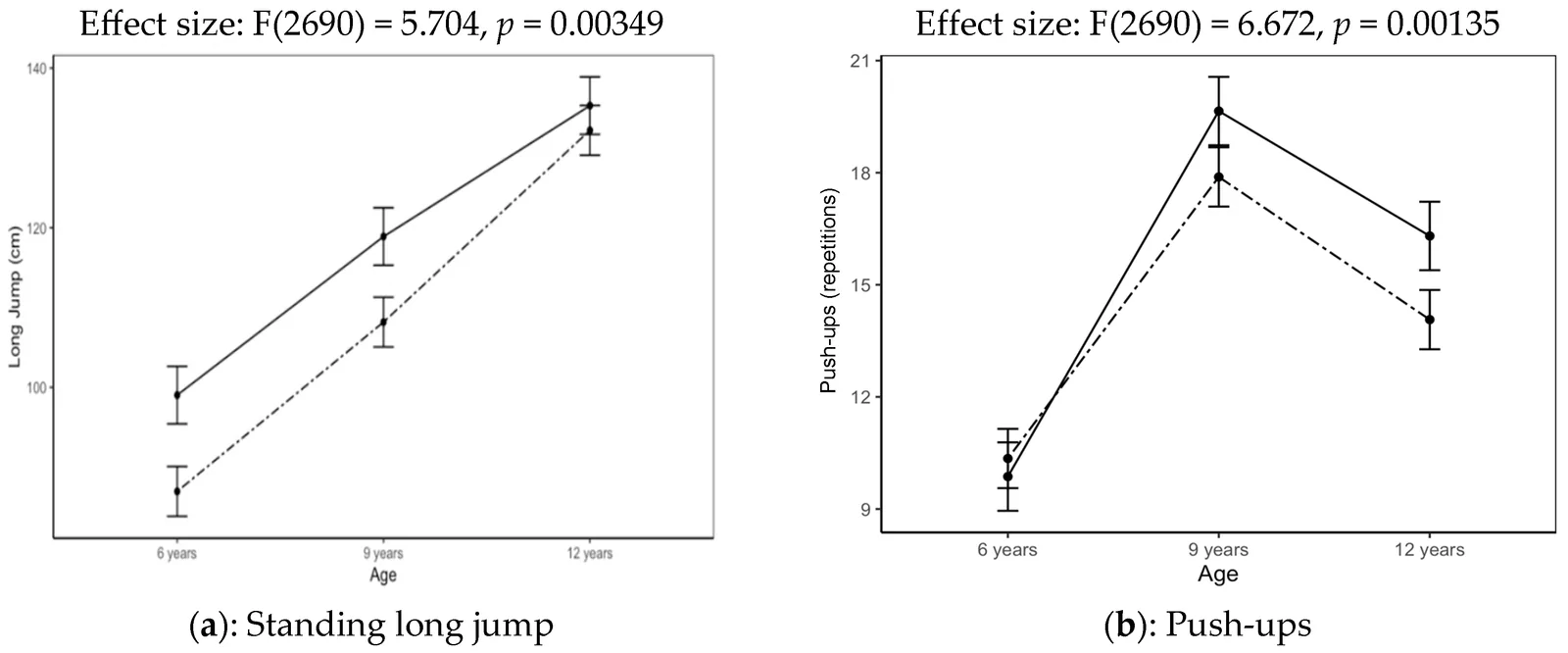

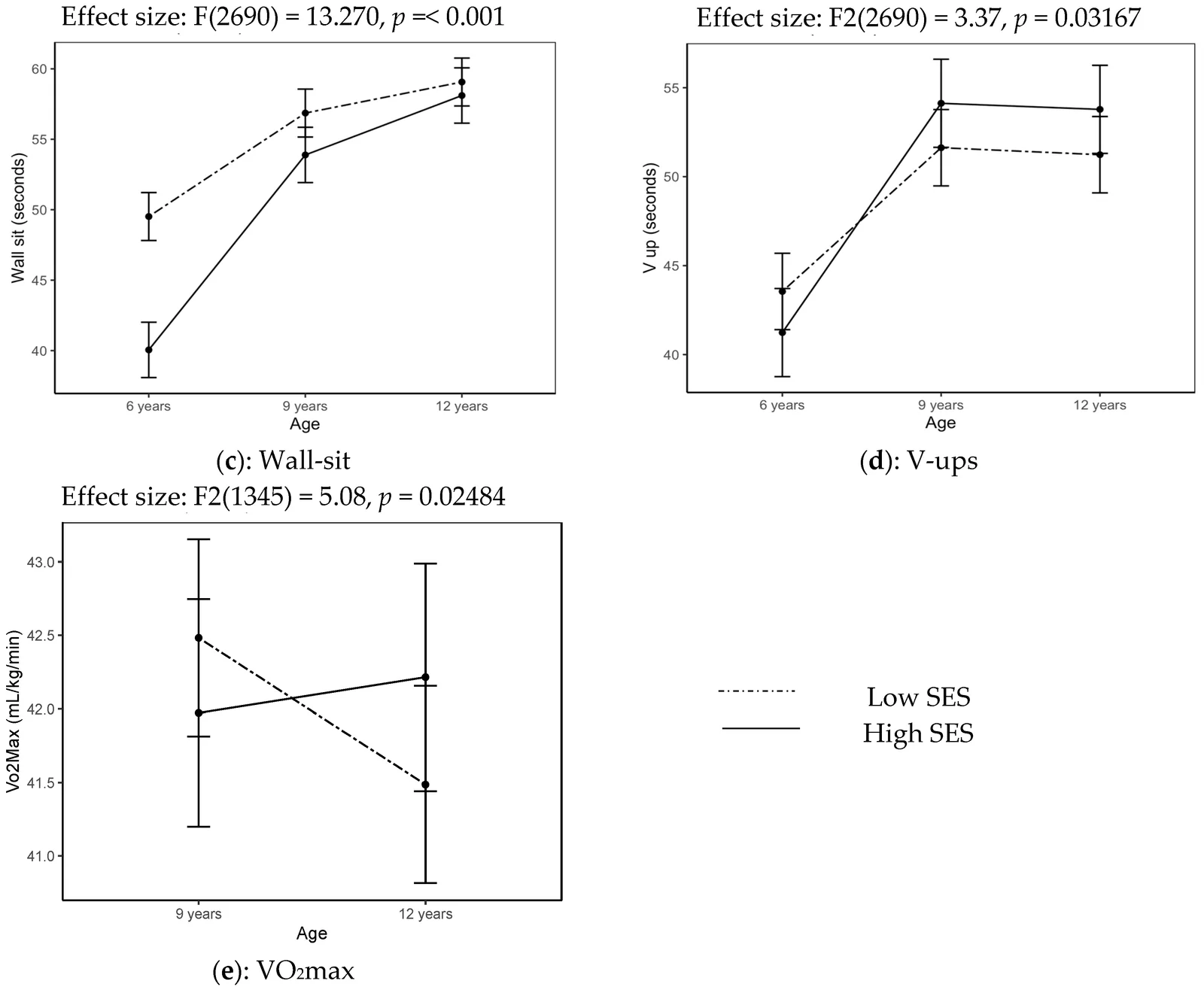
(**a**–**e**): Interaction effect of age and SES on various PF tests.

In the strength tests, being of a high SES is linked to achieving slightly higher mean scores for all MF scales in boys and girls, except for boys at age 6, where similar scores were achieved (16.80) (low SES ± 2.90; high SES ± 3.20). At the age of 9, more PACER laps are recorded for low-SES boys, although girls of a high SES achieved slightly more PACER laps compared to the low-SES group. All participants of a high SES achieved slightly more PACER laps than those of a low SES at the age of 12. Both SES groups showed an incline in strength from ages 6–9, whereafter a decline is observed at the age of 12 for boys. From ages 6–9, girls from both SES groups seems to experience a plateau in strength, whereafter a decline in strength is also seen at the age of 12 years old. Significant interaction effects according to age, SES and gender are highlighted in Tables 3–5 and illustrated by Figures 1 and 2.

Table 2 portrays similar descriptive statistics, also divided by gender in each SES group as in Table 1, but more specifically for each strength and aerobic sub-component. Increases in muscular strength in both SES groups were found for long jump and wall-sitting from ages 6–12. Boys and girls of a low SES achieved significantly (*p* < 0.01) shorter jumping distances at all ages, except for boys at the age of 12 (*p* = 0.45). At younger ages (6 and 9 years old), the high SES group held the wall-sit position for longer, although only significant at the age of 6 (*p* < 0.01). At the age of 12, non-significant (*p* = 0.98) differences were found between the low-SES (boys = 60 s; girls = 59 s) and high-SES (boys = 59 s; girls 57 s) groups in the wall-sit tests. Both genders show significant decreases in push-ups from the age of 9 (*p* < 0.01). In boys, both low (21 to 18 push-ups) and high (22 to 19 push-ups) SES groups decreased in push-up performance from the age of 9–12. Girls showed similar declining trends in performing modified push-ups (low SES = 15 to 10; high SES = 17 to 14) from the ages of 9–12. Similar trends are also observed for sit-ups where both boys (low SES = 17 to 11; high SES = 21 to 15) and girls (low SES = 15 to 8; high SES = 18 to 13) decreased significantly (*p* < 0.01) in repetition output from the age of 9. The low-SES (46 s) group of boys outperformed the high-SES (40 s) group for V-ups at the age of 6; however, this was non-significant. Thereafter, a plateau (similar or slight differences) is observed for V-ups at older ages in boys. In general, boys performed better than girls in aerobic endurance (VO_2_max) across both SES groups and time points (*p* < 0.01).

The low-SES (44.2 mL/kg/min) group achieved slightly higher VO_2_max scores at the age of 9 than boys of a high SES (43.0 mL/kg/min) with minimal differences in 2016 (low = 44.4 mL/kg/min; high = 44.6 mL/kg/min, *p* > 0.05) at the age of 12. Boys of a high SES (43.0 to 44.6 mL/kg/min) showed a larger increase in VO_2_max from age 9 to 12 compared to the low-SES boys (44.2 to 44.4 mL/kg/min). Both SES groups scored similarly for VO_2_max testing (low = 40.8 mL/kg/min; high = 40.9 mL/kg/min) at the age of 9 (*p* < 0.05). However, at the age of 12, the high-SES group (39.8 mL/kg/min) had a slightly higher average VO_2_max output compared to the low-SES group (38.5 mL/kg/min). Over time (2013–2016), VO_2_max slightly declined for girls in both high-SES (40.9 to 39.8 mL/kg/min) and low-SES (40.8 to 38.5 mL/kg/min) groups. Differences in VO_2_max were only significant between the SES groups at the age of 9 (*p* < 0.05), while gender differences were significant at the age of 9 and 12 years old (*p* > 0.05).

Initially no significant three-way interaction effects were found for age, gender and SES. Table 3 reports the interaction effects and significance of the three different two-way interaction models for all muscular and aerobic fitness tests, i.e., age and gender effect, age and SES effect and gender and SES effect. For gender and SES effects, no significant interaction effects were found for any of the PF tests. 

Table 4 displays the descriptive statistics and practical significance of differences for the age and SES interaction effect as reported in Table 3 for long jump, push-up, wall-sit, V-up and VO_2_max. These two-way interactions are also graphically displayed in Figure 2a–e No interaction effect for age and SES was found for sit-ups.

Moderate practical significant effects were found in SLJ distances for ages 6 (high SES = 38.99 cm; low SES = 34.24 cm, *p* < 0.01) and 9 (high SES = 46.81 cm; low SES = 42.59 cm, *p* < 0.01). Children of a high SES performed better in long jumping at all ages, and these differences were of medium practical significance at the ages of 6 and 9 (Figure 2a). Small practical significant differences were found in push-ups at the ages of 9 (high SES = 14.07; low SES =17.89, *p* = 0.05) and 12 (high SES =16.31; low SES = 19.65, *p* < 0.01). In push-ups, children of a high SES performed slightly poorer than those of a low SES at the age of 6 (Figure 2b). The low-SES group outperformed the high-SES group from the age of 9 to 12 years old, with differences also showing moderate practical significance (Table 4). A decline in the number of push-ups in both SES groups is also evident from the age of 9 to 12 years (Figure 2b).

For wall-sitting a large practical effect is observed at 6 years (high SES = 40.05; low SES = 49.52, *p* = 0.00) with a smaller but non-significant effect at 9 years old (high SES = 53.89; low SES = 40.05, *p* = 0.22). The low-SES group performed better in wall-sitting (Figure 2c) at all ages (2010–2016), although the group differences narrowed from 9 years, where they were still practically significant (d = 0.27) but not at 12 years (d = 0.09).

Figure 2d illustrates that the high-SES group was outperformed by the low-SES group at the age of 6 in V-ups. However, as children aged, contradictory trends were observed where the high-SES group outperformed the low-SES group at older ages (9 to 12 years). Despite these trends, no practical differences in V-ups were observed at any age.

Similar trends can be observed in Figure 2e for VO_2_max. No practical differences (d = 0.13) were found in VO_2_max between the high-SES (41.97 mL/kg/min) and low-SES (42.48 mL/kg/min, *p* = 0.73) groups at the age of 9, where the low-SES group achieved higher mean scores. However, at age 12, a small practical, yet non-significant, effect revealed a shift where the high-SES group (42.22 mL/kg/min) outperforms the low-SES group (41.49 mL/kg/min) (*p* = 0.50). The low-SES group showed a relatively steep decline in VO_2_max values (2013 = 42.48 mL/kg/min; 2016 = 41.49 mL/kg/min) over the 3-year time period, in contrast to the high-SES group who showed a steady increase in VO_2_max during the same period.

**Figure 3:**
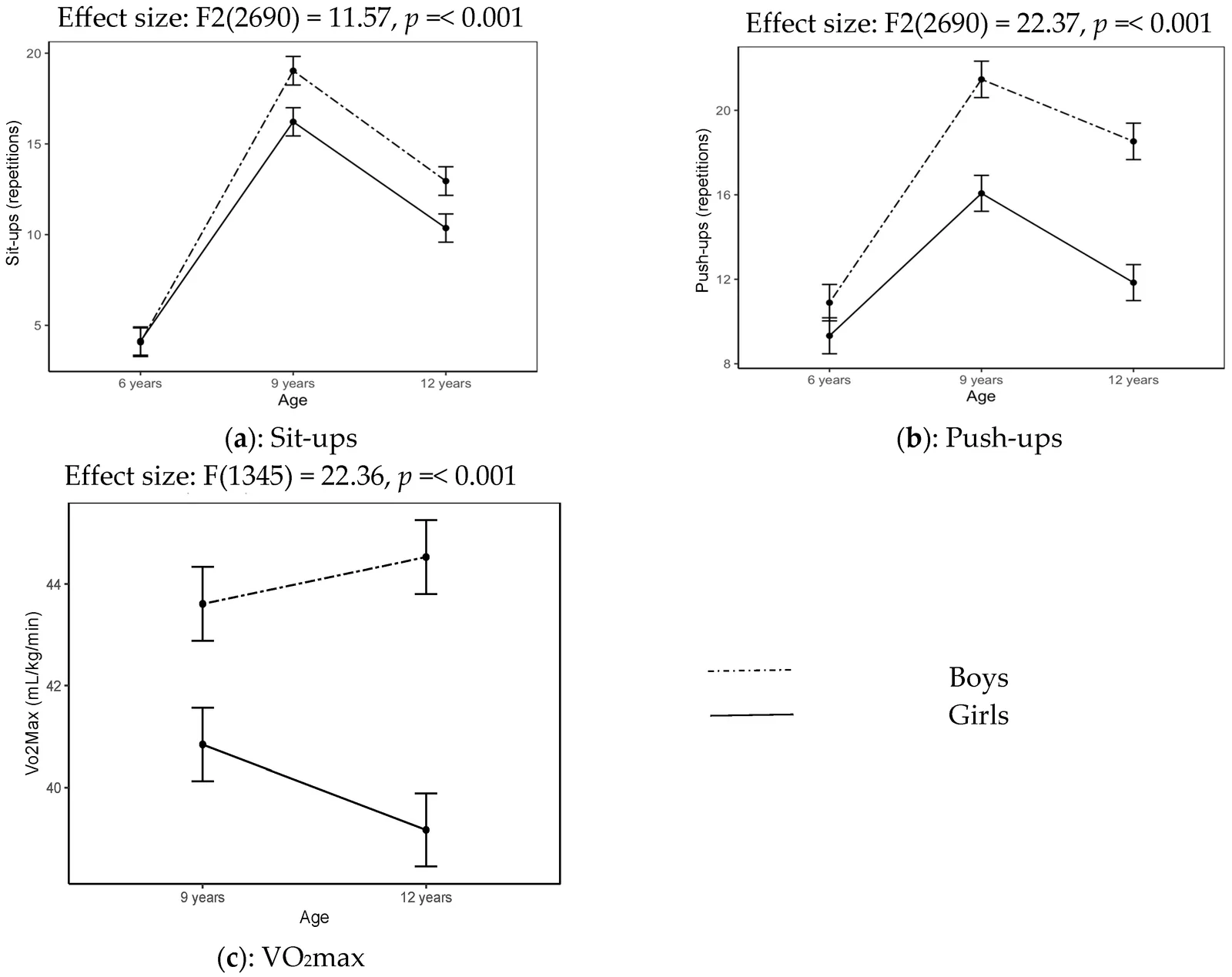
Interaction effects of age and gender on muscular and aerobic fitness tests.

Table 5 displays the significant gender and age interaction effects reported for sit-ups, push-ups and VO_2_max (Table 3) with a graphical display of the trends in Figure 3a–c. Gender differences are noted in sit-ups (boys = 4.07 reps; girls = 4.13 reps, d = −0.02), as boys performed better than girls from 9 years (d = 0.65; d = 0.60). Boys (19.03 to 12.95 reps) and girls (16.22 to 10.36 reps) also decreased in sit-up performance from 9 to 12 years. The push-up performance of boys was better at all ages (d = 0,31; d = 1.05; d = 1.30) compared to girls. Declines in the number of push-ups are, however, noticed for both gender groups from 9 years old (boys = 21.47 reps; girls = 16.07 reps) to 12 years old (boys = 18.53 reps; girls = 11.84 reps). Figure 3c shows noticeable gender differences at the age of 9 with widening effects over time (d = 0.78, d = 1.51), where boys (43.61 mL/kg/min) achieved better VO_2_max scores compared to girls (41.76 mL/kg/min). Inverse effects are noticeable with boys (43.61 to 44.53 mL/kg/min) achieving higher VO_2_max values at age 12 compared to girls (40.85 to 39.17 mL/kg/min) who show a decline over the 3-year time period.

## 4. Discussion

This study aimed to determine differences to global trends in the PF profiles of South African primary school children by using longitudinal data. Possible risk factors such as age, gender and SES in the fitness profiles were investigated to identify the need for early prevention strategies. None of the strength or CRF variables yielded three-way interactions. However, two-way interactions between age and gender, as well as age and SES, were found for several of the fitness variables, depending on age.

### 4.1. Comparing Criteria Reference Standards (CRSs) and Interaction Effects of Age, Gender and SES on MF and CRF

#### 4.1.1. CRSs and Interaction Effects of Age and SES for V-Ups and Wall-Sitting

Results from the study found that wall-sit and V-ups were both influenced by age and SES. Apart from inclusions in test batteries, to our knowledge, no percentile or cut-offs have specifically been reported for the V-ups and wall-sitting tests in children and adolescents to be used for comparison purposes as a stand-alone CRSs to assess PF levels. The Fitnessgram only includes a trunk lift, although similar to the V-up tests, it is not tested by measuring time held in the position but rather the distance the chest is lifted off the ground with no leg lift included [18]. These tests, which represent muscular endurance, yielded inconclusive findings although muscular endurance does not seem to be the strongest and most inclusive indicator of PF but rather muscular strength tests are.

#### 4.1.2. CRSs and Interaction Effects of Age and SES for Standing Long Jump

SLJ is an indicator of general MF in youth and is one of the most utilized MF tests globally that assesses lower body strength and, more specifically, explosive strength [47]. Thomas et al. [48] reported age- and gender-specific PF percentile norms for children aged 6–18, including SLJ, based on data from seven European countries that revealed poorer performance than our participants. The 50th percentile values represent an average performance for boys of 124 cm at age 6, 133 cm at age 9 and 163.5 cm at age 12. Both low-SES (6 years = 91.4 cm; 9 years = 112.3 cm; 12 years = 137.7 cm) and high SES (6 = year = 106.7 cm; 9 years = 127.9 cm; 12 years = 140.1) groups from the current study performed below the reported 50th percentiles at all ages. The 50th percentiles for females, denoted as 115.5 cm at age 6, 122.5 cm at age 9 and 150.5 cm at age 12 [48], compared to 83.8 and 91.4 cm (6 years), 104.1 cm and 111.7 cm (9 years), 124.1 cm and 132.3 cm (12 years) of our group, also show that both low-SES and high-SES female groups performed below average at all three ages. Similar results were found when comparing the 50th percentile cut-offs reported by Tomkinson et al. [17] and Kolimechkov et al. [49]. Cut-points by Tomkinson [17] and Kolimechkov et al. [49] created comprehensive percentile scores for PF tests such as the SLJ, based on existing European data. In some cases, the low-SES groups significantly underperformed, falling below the 10th percentile, which is worrying. Compared to a South African study by Africa et al. [50] from the Western Cape Province, results indicated that boys (high SES = 120.6 cm, low SES = 113.0 cm compared to high SES = 106.7 cm, low SES = 91.4 cm) and girls (high SES = 108.0 cm, low SES = 105.2 cm compared to high SES = 91.4 cm, low SES = 83.8 cm) from this study achieved shorter jumping distances for SLJ at the age of 6. This comparison also yields differences in the South African child population based on the different regions in S.A.

A significant two-way interaction effect was found for the SLJ regarding age and SES (*p* = 0.003). Contrary to findings from Africa et al. [50], in 6 year-old children, no effect of SES was found for SLJ. However, the current study revealed higher performance in high-SES children in SLJ at all ages with practical significance at the ages of 6 and 9 years. Findings by Smith et al. [15], based on 9-year-olds from the Eastern Cape in S.A, reported no significant differences between SLJ distances of SES groups. Africa et al. [50] reported that children of a low SES performed significantly better in dynamic strength of the trunk and upper limbs compared to children of a higher SES, however, no SES differences were found for lower limb strength assessed with SLJ. These differences can be attributed to children of a high SES being more prone to attending organised sports activities as opposed to children of a low SES who would play more simple and spontaneous unstructured games. Through this repetitive play of spontaneous games, motor learning would take place which can give children of a low SES an advantage [50]. However, this is contradictory to our findings as children of a high SES performed better at all ages. The way children of different SESs spend their free time can influence how they learn and develop their physical strength.

Cut-points by Castro-Piñero et al. [12] for SLJ are further linked to a healthy cardiovascular profile for children (6–9 years old) and adolescents (12–16 years old). These cut-points are 104.5 cm for boys and 81.5 cm for girls in the 6–9 age group [12]. In the current study, the average SLJ distances for children aged 6–9 were reported as ranging from 91.4 cm (low SES) to 127.9 cm (high SES) for boys and 83.1 cm (low SES) to 111.7 cm (high SES) for girls. The cut-points set by Castro-Piñero et al. [12] for the adolescent group are 140.5 cm for boys and 120.5 cm for girls. According to the current findings, boys and girls reached an average jumping distance of 137.7 cm (low SES) to 140.1 cm (high SES) and 124.1 cm (low SES) to 132.3 cm (high SES) at the age of 12, respectively. Only girls reached these cut points at ages 6–12. Based on these cut-points by Castro-Piñero et al. [12] that identify cardiovascular disease (CVD), South African boys are at risk and likely to develop CVD 2 years later. Our study found that low SES hinders SLJ distances, leading to health risks, especially in boys.

#### 4.1.3. CRSs and Interaction Effects of Age, SES and Gender for Push-Ups

The current study uses the BOT-2, which gives the option of modified push-ups. The Fitnessgram includes full push-ups as part of MF testing, which is a different protocol to the BOT-2 [18], therefore comparison between these two tests was not possible as modified push-ups are easier to complete than 90° push-ups. Cut-points by Niessner et al. [51], based on a German sample of children, included push-ups as part of PF testing, however, again these protocols differed as children had to do a push-up and hold the position while using one hand to touch the other before lowering down. This addition was added to specifically assess muscular endurance. Significant SES effects have been found between the ages of 9 and 12, where children of a high SES performed slightly poorer than those of a low SES. Age and gender also showed significant interaction effects (*p* < 0.001), where boys outperformed girls from 6–12 years, with practical significance in all age groups. In agreement, Gherghel [52] also reported boys performing better in push-ups between the ages of 6 and 11 years old compared to girls. According to the Fitnessgram cut-points, gender differences can only be expected from the age of 11 years [18]. Niessner et al. [51] reported maximal arm strength for girls at the age of 11. However, the cut-point by Niessner et al. [51] indicates fairly similar outputs for boys and girls on the 50th percentile, with boys experiencing increases after 11 years old and girls a plateau. Although no specific comparisons could be made, South African children followed global trajectories of muscular strength tests, where boys seem to have greater upper body strength. However, it may be necessary to establish universal and nationally accepted protocols to assist in monitoring the arm strength of children at a national level. Girls may need more monitoring and intervention at an early age since gender differences are noticeable as early as age 6, even though these differences typically emerge at age 11. Age effects emerged from the age of 9 in both boys and girls, with performance showing a declining trend onwards.

#### 4.1.4. CRSs and Interaction Effects of Age and Gender for Sit-Ups

A significant two-way interaction effect was reported for sit-ups with regard to SES and gender. Gender- and age-specific cut-offs by Tomkinson et al. [17] were based on a similar 30 s protocol from the Eurofit test battery which included data from 23 countries for children aged 9–17 years old. A minimum of 17 and 21 sit-ups are required to meet the 50th percentile for boys and 17 and 19 sit-ups for girls at ages 9 and 12, respectively. Boys of low (17 reps) and high (21 reps) SESs score at or above the 50th percentile at age 9, with decreasing trends at age 12 where both low-SES (11 reps) and high-SES (15 reps) groups fell below the 50th percentile. Girls of a high SES (18 reps) scored slightly above the 50th percentile at age 9 while the low-SES group (15 reps) fell below the 50th percentile. At the age of 12, both SES groups (low = 8 reps; high = 13 reps) fell below the 50th percentile. Similar findings were observed when using the Fitnessgram criteria to classify the groups into the HFZ. According to the Fitnessgram [18], boys and girls at the age of 6 should be able to perform at least three sit-ups to be deemed in the HFZ. At the age of 9, 9 sit-ups are required for boys and girls and, at the age of 12, boys are required to do at least 18 sit-ups [18]. Boys of both low and high SESs reached these cut-offs at ages 6 and 9 but, at the age of 12, low- and high-SES groups of boys could only perform an average of 11 and 15 sit-ups, not reaching these cut-points. Similarly for girls, 8 and 13 sit-ups were reported for the low- and high-SES groups, respectively, thus not meeting the HFZ criteria of 18 repetitions at the age of 12. The Fitnessgram does not rely on percentiles, but rather uses a CRS which is linked to specific health outcomes. This approach provides a minimum standard to achieve to be classified in the HFZ [4]. Gender had a significant effect over time with boys performing better than girls at most ages, except at the age of 6 (*p* < 0.001). These differences agree with global literature on boy–girl differences [4,17,53]. These gender advantage can be attributed to age-related increases in muscle strength in boys while girls only experience an increase in abdominal muscular strength up to the age of 11–12 years old [53].

Comparing sit-up protocols across studies is challenging due to differences in arm and leg positions, trunk flexion and timing intervals [4,29,51]. The Fitnessgram uses curl-ups [18], which are considered safer and differ from the protocols used by Niessner et al. [51] and the BOT-2 protocols [44]. These inconsistencies limit the ability to directly compare results across studies, making it difficult to draw definitive conclusions. Therefore, the adoption of a universally standardised testing protocol is recommended to ensure consistency and comparability in future research.

#### 4.1.5. CRSs and Interaction Effects of Age, SES and Gender for VO_2_max (CRF)

Globally, the 20 m shuttle run test is deemed a valid and reliable field-based test to use as a CRS for aerobic capacity [23]. According to the results from the current study, boys of a low SES achieved a VO_2_max of 44.2 mL/kg/min and 44.4 mL/kg/min at ages 9 and 12, respectively, which places them in the HFZ according to the Fitnessgram cut-points [18]. Based on these norms, boys can be classified into the HFZ at age 10 when achieving a VO_2_max of 40.2 mL/kg/min or above and 40.3 mL/kg/min or above at age 12 [18]. For girls, VO_2_max values of 40.2 mL/kg/min and 40.1 mL/kg/min are required at ages 10 and 12 to be classified into the HFZ for CRF [18]. Our study indicated that both girls of high (40.9 mL/kg/min) and low (40.8 mL/kg/min) SESs achieved the HFZ classification at the age of 10. However, at the age of 12, both low-SES (38.5 mL/kg/min) and high-SES (39.8 mL/kg/min) groups were classified into the needs improvement (NI) zone as they did not achieve the desired PF levels of 40.1 mL/kg/min. These results are in accordance with worldwide literature, which indicates that girls have lower PF levels, displaying that children from the NWP are also at risk [25,54]. However, when comparing CRSs for aerobic capacity, as indicated by Tomkinson et al. [24], results indicate health risks when thresholds of 42 and 35 mL/kg/min for boys and girls, respectively, are not reached. According to the Ruiz [7] standards, which indicate a healthy cardiovascular profile based on aerobic capacity, a value below these recommended cut-points should raise a red flag and be deemed a health risk for 8–17-year-olds. Results from the current study show that both SES and gender groups meet these thresholds at the age of 9. However, at the age of 12, only the boys meet the criteria, with girls aged 12 urgently classified as at risk. However, these levels still require monitoring for both boys and girls.

Significant two-way interactions were found for VO_2_max with regard to age and SES (*p* = 0.025) with practical significance at the age of 12. The disparities between SES groups were mostly evident in girls where the high-SES group achieved higher CRF levels than those of a low SES. These effects were also widening and greater at the age of 12 compared to 9-year-olds as is evident from the results in Figure 3c. The Global Physical Activity Report Card Grades for Children and Adolescents report that PA is hampered by economic challenges in South African youth [23], which may explain the impact on PF levels since PA is a direct influencer of PF. According to the Cooper Institute (Fitnessgram cut-offs), this is expected as younger boys and girls achieve fairly similar results. However, as maturation occurs, boys and girls will have unique developmental trajectories, which will affect their PF standards [18]. As boys mature, a slight increase in VO_2_max can be expected, compared to a decrease in girls which can be attributed to increases of body fat during puberty [55]. This is also confirmed by results from the present data where significant interaction effects were reported between age and gender (*p* < 0.001), with practical significance from 9 years old. The study by Smith et al. [15] also confirms these findings. Therefore, it is crucial to consider maturity, growth and body composition as influencing factors of PF during childhood and adolescence as boys have greater aerobic capacity from 9 years old [55].

### 4.2. Utilising International CRSs in South African Child Populations

It appears that South African primary school children have PF trajectories that are consistent and comparable to those observed globally. The strength and aerobic endurance of South African children are not different from worldwide trends. Global findings furthermore confirm our findings that boys have higher muscular strength and CRF levels than girls and that PF levels increase with age. However, some gender and SES groups did not reach these universal cut-points in selected strength and aerobic tests, which necessitates the need for a closer look at possible reasons behind these findings. Also, the need for targeted intervention strategies, particularly for “at-risk” groups such as girls, older children and those from low SES backgrounds, rather than simply relying on national cut-off thresholds is evident from the findings.

The Global Matrix 4.0 Physical Activity Report Card Grades for Children and Adolescents [23] stresses the importance for countries to develop their own strategies and action plans to promote PA according to their unique context. Fitness indicators that were used in this study included the sit-and-reach flexibility test, handgrip strength, sit-ups and the 20 m shuttle run test. Such action plans and strategies will counter poor PF levels and obesity to ultimately contribute to achieving the vision of the Global Action Plan by 2030 [6]. The Global Matrix 4.0 Physical Activity Report Card Grades for Children and Adolescents presents a universally inclusive impression of PA from a global stance by including data from 57 countries for children ages 5 to 17 years old, also including South African statistics. S.A scored a B- on PF, indicating that children in this country fell within the 60–66th percentile for PF tests of 5–17-year-olds, according to cut-points provided by a review study of Tomkinson and co-workers. These results included national findings based on national report card studies, which included available data from within their country. These studies mostly included regional-specific data based on S.A, as no national studies are currently available to assess overall PF in S.A [23,35]. Similar findings were reported on the active play and overall PA categories. The Healthy Active Kids South African Report Card (HAKSA) of 2016 reported that although primary school children from Limpopo Province of S.A achieved high levels of PA, they still display poor CRF levels when measuring VO_2_max [25]. Castro-Piñero et al. [12] noted that MF is independent of CRF which necessitates additional monitoring of strength as a health marker since poor levels of MF are related to greater cardiovascular health risks 2 years later. For MF testing, one of the major strength tests identified by the literature as an indicator of CVD is the SLJ. While SLJ primarily assesses lower body strength, it also correlates with upper body strength, underscoring the need to include this PF test in all PF assessments.

It is recommended that monitoring of PF trends should be prioritised worldwide [8]. Kaster et al. [9] furthermore stress the importance of monitoring PF in LMICs to determine authentic global trajectories since SES might be a contributing factor to differences in these fitness levels [8]. Although no clear SES differences emerged from our findings and some cut-points are available from the literature and test batteries for sit-ups, South Africa must recommend universally acceptable and comparable national protocols. Additionally, push-up protocols differ between testing instruments, further emphasising the need for standardised protocols. The development and implementation of a fitness protocol that can be used nationally with annual testing of children in the seven primary school years is recommended. Such testing should ideally be performed by physical education teachers. Fitness testing of a large national sample of children from all provinces in S.A over a longer time throughout adolescence will provide a clearer and more detailed picture than the current data. This will also allow for better tracking of PF levels throughout childhood and adolescence for monitoring purposes. A further recommendation is standard universal protocols for assessing various fitness tests such as push-ups and sit-ups to enhance global and national comparability. Developing unique national PF cut-points in future might be necessary once data from all eight provinces yield results.

### 4.3. Strengths and Limitations

The strength of the study lies in its unique design as one of few studies that aimed to find, with longitudinal data, if South African primary school children reach international PF benchmarks to determine health risks. This study provides a comparison of South African children’s PF levels with international standards, providing valuable insights into how these children measure up against global CRSs specific to age, gender and SES groups to ensure early intervention. Most studies focus on CRF standards but do not include all PF parameters and are not specific to the context of S.A. The current study used VO_2_max as an indirect predictor of CRF via the 20 m shuttle run test, which is the most utilised international fitness test, including the Fitnessgram. The large, random, stratified study sample and the longitudinal nature, inclusive of children from all backgrounds, strengthens the applicability of the study. However, the study also had limitations to be acknowledged. Although the study used a big sample, it still only represented one province in S.A, and the high dropout rate over the 7-year period may influence generalisability. A further limitation is that testing conditions at all schools were not always ideal because some low-SES schools lacked sports facilities to conduct fitness tests in optimal conditions.

## 5. Conclusions

This study utilised global protocols and cut-points to assess and compare PF levels in South African children. The study confirmed that South African primary school children achieved several of the universally recommended cut-points for CRF and PF standards. Findings from the study, however, highlight the importance of acknowledging the influence of age, gender and SES on PF when addressing and promoting PF levels in primary school children through the development of specific health promotion interventions.

The continued use of universal standards and incorporating specific MF tests such as SLJ, push-ups and sit-ups along with CRF are recommended to provide a good understanding of current but also changing trends in PF and in compiling comprehensive intervention programs tailored to South African children. This approach will aid in identifying health risks, while using globally standardised cut-points will further assist with monitoring youth. Further national research on CRSs is crucial to inform targeted interventions needed to bridge these disparities and enhance overall PF levels in children from various backgrounds. Continued exploration of national PF cut-points based on gender, age and SES specific to SLJ, sit-ups and push-ups is needed to ensure comparable protocols. Annual implementation and monitoring of fitness tests specific to age, SES and gender should be conducted by trained professionals in the field of child health and wellness. In addition, upskilling of physical education teachers is necessary to monitor children’s physical health continuously within the school context to ensure early intervention. Policymakers should use these results to make informed decisions on timely implementation of prevention strategies. Based on the current findings, targeted PF intervention with a focus on girls, older children from the age of 9 and children from low-SES backgrounds should be prioritised.

## Figures and Tables

**Table 1:** Descriptive characteristics of sociodemographic, anthropometric and physical fitness indicators at baseline and follow-up time point measurements (*N* = 349).

Variables	Boys (*n* = 165)						Girls (*n* = 184)					
	Low SES (*n* = 83)			High SES (*n* = 82)			Low SES (*n* = 118)			High SES (*n* = 66)		
	M ± SD	Min	Max	M ± SD	Min	Max	M ± SD	Min	Max	M ± SD	Min	Max
**Biographic and Anthropometric**												
Age (years)												
6 years old	6.76 ± 0.48	6.00	7.80	6.90 ± 0.55	6.10	7.80	6.70 ± 0.44	6.00	7.80	6.88 ± 0.54	6.10	7.70
9 years old	9.84 ± 0.38	8.99	10.67	10.04 ± 0.35	9.32	10.68	9.77 ± 0.35	9.00	10.68	10.01 ± 0.36	9.24	10.64
12 years old	12.84 ± 0.39	11.89	13.68	13.05 ± 0.35	12.34	13.68	12.78 ± 0.36	12.03	13.68	13.02 ± 0.36	12.26	13.65
Stature (cm)												
6 years old	116.99 ± 4.69	102.10	130.30	124.50 ± 5.89	112.20	141.70	117.06 ± 4.94	107.90	128.50	122.33 ± 6.01	109.70	134.70
9 years old	132.33 ± 4.99	117.00	147.10	139.77 ± 6.60	123.20	161.00	134.03 ± 6.00	121.40	151.00	138.91 ± 7.84	113.00	155.00
12 years old	147.78 ± 7.15	131.90	169.40	157.24 ± 8.34	139.40	178.80	151.98 ± 6.38	136.70	168.80	157.35 ± 7.37	143.60	185.90
Body mass (kg)												
6 years old	20.67 ± 2.52	16.40	30.50	26.76 ± 5.73	17.20	45.90	20.74 ± 3.06	15.08	29.70	24.18 ± 4.97	16.40	42.80
9 years old	28.58 ± 5.26	19.60	55.90	37.31 ± 9.86	22.60	65.60	30.13 ± 6.88	21.70	55.80	36.09 ± 8.78	22.20	64.70
12 years old	39.05 ± 9.31	23.10	84.80	52.35 ± 14.17	30.80	100.80	44.17 ± 10.05	28.80	78.50	52.19 ± 12.37	36.90	95.20
BMI (Kg/m^2^)												
6 years old	15.18 ± 1.46	12.90	22.90	16.50 ± 2.70	12.90	26.80	15.10 ± 5.00	12.70	20.70	16.10 ± 2.50	12.90	24.00
9 years old	16.27 ± 2.37	12.90	25.83	19.00 ± 3.90	13.30	30.80	16.70 ± 3.00	13.10	27.40	18.50 ± 3.80	14.20	29.70
12 years old	17.81 ± 3.23	14.00	32.00	21.10 ± 4.70	14.90	36.50	19.00 ± 3.60	13.90	33.70	21.10 ± 4.40	16.10	34.50
∑ skinfolds (mm)												
6 years old	12.40 ± 3.40	8.20	27.00	16.00 ± 8.00	8.00	47.00	15.00 ± 5.00	8.00	38.00	19.00 ± 7.00	9.00	40.00
9 years old	13.30 ± 7.30	7.00	57.50	22.00 ± 11.00	8.00	53.00	18.00 ± 8.00	8.00	46.00	25.00 ± 11.00	12.00	54.00
12 years old	15.10 ± 11.40	7.50	78.20	25.00 ± 15.00	9.00	76.00	22.00 ± 10.00	9.00	60.00	28.00 ± 14.00	14.00	75.00
Body fat (%)												
6 years old	14.60 ± 3.30	8.70	27.80	18.00 ± 6.00	10.00	39.00	14.00 ± 4.00	8.00	29.00	17.00 ± 6.00	8.00	32.00
9 years old	18.10 ± 6.70	5.00	38.40	23.00 ± 8.00	7.00	42.00	19.00 ± 8.00	5.00	44.00	24.00 ± 9.00	10.00	47.00
12 years old	17.30 ± 7.10	7.60	41.80	22.00 ± 9.00	5.00	45.00	23.00 ± 8.00	10.00	46.00	27.00 ± 8.00	11.00	48.00
**Physical Fitness**												
Strength (SS)												
6 years old	16.80 ± 2.90	9.00	23.00	16.80 ± 3.20	9.00	23.00	15.30 ± 3.40	6.00	22.00	16.30 ± 3.70	8.00	29.00
9 years old	17.70 ± 3.00	8.00	24.00	18.60 ± 3.00	10.00	27.00	15.90 ± 3.00	7.00	22.00	16.90 ± 3.60	10.00	25.00
12 years old	13.90 ± 2.20	7.00	18.00	14.50 ± 2.50	8.00	21.00	13.10 ± 2.10	7.00	19.00	14.60 ± 3.20	8.00	22.00
PACER (laps) ^												
9 years-old	27.00 ± 15.00	4.00	74.00	24.00 ± 13.00	5.00	65.00	17.00 ± 9.00	2.00	59.00	19.00 ± 9.00	7.00	45.00
12 years-old	37.00 ± 16.00	7.00	82.00	39.00 ± 19.00	8.00	83.00	20.00 ± 10.00	6.00	58.00	25.00 ± 17.00	5.00	80.00

BMI = Body mass index; M = Mean; Max = Maximum value; Min = Minimum value; SD = Standard deviation; SES = Socioeconomic status; ∑ skinfolds = Sum of sub-scapular + triceps skinfolds; SS = Scale score; ^ = Not measured in 2010.

**Table 2:** Descriptive characteristics of muscular strength and aerobic endurance sub-tests according to gender and SES over three time points (*N* = 349).

Variables	Boys (*n* = 165)						Girls (*n* = 184)					
	Low SES (*n* = 83)			High SES (*n* = 82)			Low SES (*n* = 118)			High SES (*n* = 66)		
	M ± SD	Min	Max	M ± SD	Min	Max	M ± SD	Min	Max	M ± SD	Min	Max
Long jump (cm)												
6 years old	91.40 ± 20.30	43.20	134.60	106.70 ± 15.20	139.70	168.66	83.08 ± 15.20	50.80	134.62	91.40 ± 15.20	50.80	134.00
9 years old	112.30 ± 21.49	75.01	172.72	127.86 ± 22.63	76.99	177.04	104.10 ± 17.80	58.40	149.90	111.70 ± 25.40	53.30	165.10
12 years old	137.74 ± 16.37	91.44	177.80	140.13 ± 23.52	79.50	195.58	124.06 ± 17.16	76.20	168.00	132.27 ± 20.43	69.12	197.12
Push-up (reps)												
6 years old	12.00 ± 6.00	0.00	26.00	10.00 ± 5.00	0.00	25.00	9.00 ± 6.00	0.00	20.00	10.00 ± 5.00	0.00	17.00
9 years old	21.00 ± 5.00	10.00	33.00	22.00 ± 5.00	11.00	40.00	15.00 ± 5.00	0.00	28.00	17.00 ± 6.00	5.00	30.00
12 years old	18.00 ± 7.00	2.00	35.00	19.00 ± 6.00	6.00	33.00	10.00 ± 5.00	0.00	26.00	14.00 ± 7.00	1.00	27.00
Sit-up (reps)												
6 years old	3.00 ± 3.00	0.00	11.00	5.00 ± 4.00	0.00	17.00	2.00 ± 3.00	0.00	12.00	6.00 ± 4.00	0.00	15.00
9 years old	17.00 ± 6.00	0.00	32.00	21.00 ± 6.00	2.00	50.00	15.00 ± 6.00	0.00	28.00	18.00 ± 5.00	0.00	32.00
12 years old	11.00 ± 4.00	0.00	19.00	15.00 ± 7.00	2.00	60.00	8.00 ± 5.00	0.00	21.00	13.00 ± 5.00	0.00	26.00
Wall-sit (s) *												
6 years old	49.00 ± 15.00	10.0	60.00	40.00 ± 20.00	6.00	60.00	50.00 ± 15.00	10.00	60.00	40.00 ± 19.00	8.00	60.00
9 years old	58.00 ± 7.00	23.00	60.00	54.00 ± 12.00	14.00	60.00	56.00 ± 10.00	20.00	60.00	54.00 ± 14.00	6.00	60.00
12 years old	60.00 ± 3.00	42.00	60.00	59.00 ± 4.00	34.00	60.00	59.00 ± 6.00	18.00	60.00	57.00 ± 9.00	10.00	60.00
V-up (s) *												
6 years old	46.00 ± 17.00	7.00	60.00	40.00 ± 19.00	0.00	60.00	41.00 ± 20.00	0.00	60.0	42.00 ± 18.00	0.00	60.00
9 years old	53.00 ± 12.00	14.00	60.00	56.00 ± 11.00	7.00	60.00	50.00 ± 17.00	0.00	60.0	52.00 ± 14.00	3.00	60.00
12 years old	53.00 ± 12.00	10.00	60.00	54.00 ± 11.00	10.00	60.00	50.00 ± 13.00	20.00	60.0	53.00 ± 14.00	5.00	60.00
VO_2_max (mL/kg/min) ^												
9 years old	44.20 ± 5.20	35.70	60.30	43.00 ± 4.40	35.80	57.30	40.80 ± 2.90	35.70	54.60	40.90 ± 3.20	36.30	50.90
12 years old	44.40 ± 5.50	33.30	59.70	44.60 ± 6.80	33.20	60.30	38.50 ± 3.60	33.20	51.30	39.80 ± 6.00	32.60	59.10

* 60 s was the maximum requirement to score full points for the wall-sit and V-up sub-tests. cm = Centimeters; M = Mean; Max = Maximum value; Min = Minimum value; reps = Repetitions; SD = Standard deviation; sec = Seconds; SES = Socioeconomic status; ^ = Not measured in 2010.

**Table 3:** Interaction effect of sociodemographic factors for the different PF tests (*N* = 349).

PF tests	Model	MS	F	*p*
Standing long jump	Age and Gender	14.31	0.04	0.96
	Age and SES	1955.00	5.70	0.00 *
	Gender and SES	704.00	2.05	0.15
Sit-ups	Age and Gender	214.40	11.57	<0.00 *
	Age and SES	43.19	2.33	0.10
	Gender and SES	7.63	0.41	0.52
Push-ups	Age and Gender	592.30	22.37	<0.00 *
	Age and SES	176.70	6.67	0.00 *
	Gender and SES	87.34	3.30	0.07
Wall-sit	Age and Gender	56.32	0.45	0.64
	Age and SES	1651.00	13.27	<0.00 *
	Gender and SES	0.026	0.00	0.99
V-ups	Age and Gender	48.44	0.26	0.77
	Age and SES	650.90	3.47	0.03 *
	Gender and SES	337.50	1.80	0.18
VO_2_max	Age and Gender	282.5	22.36	<0.00 *
	Age and SES	464.15	5.08	0.02 *
	Gender and SES	23.63	1.87	0.17

MS = Mean squared; SES = Socioeconomic status; * *p* < 0.05 = Significant.

**Table 4:** Interaction effect of age and SES of various PF tests (*N* = 349).

Year Group	Low SES (*n* = 83 + 118)					High SES (*n* = 82 + 66)					d
	EMM	SE	df	Lower CL	Upper CL	EMM	SE	df	Lower CL	Upper CL	
Standing long jump											
6 yo	86.97	1.59	873.61	82.78	91.16	99.03	1.84	873.61	94.20	103.87	−0.65 ^##^
9 yo	108.18	1.59	873.61	103.99	112.37	118.90	1.84	873.61	114.06	123.74	−0.58 ^##^
12 yo	132.20	1.59	873.61	128.01	136.39	135.29	1.84	873.61	130.46	140.13	−0.17
Push-ups											
6 yo	10.35	0.40	979.07	9.29	11.42	9.87	0.47	979.07	8.64	11.10	0.09
9 yo	17.89	0.40	979.07	16.82	18.95	19.65	0.47	979.07	18.42	20.88	−0.34 ^#^
12 yo	14.07	0.40	979.07	13.00	15.13	16.31	0.47	979.07	15.08	17.54	−0.44 ^#^
Wall-sit											
6 yo	49.52	0.867	989.71	47.23	51.80	40.05	1.00	989.71	37.41	42.69	0.85 ^###^
9 yo	40.05	1.001	989.71	37.41	42.69	53.89	1.00	989.71	51.25	56.53	0.27 ^#^
12 yo	59.06	0.867	989.71	56.78	61.35	58.11	1.00	989.71	55.47	60.74	0.09
V-ups											
6 yo	43.55	1.09	962.11	40.67	46.43	41.24	1.26	962.11	37.91	44.56	0.17
9 yo	51.63	1.09	962.11	48.75	54.51	54.13	1.26	962.11	50.80	57.45	−0.18
12 yo	51.24	1.09	962.11	48.35	54.12	53.78	1.26	962.11	50.46	57.11	−0.19
VO_2_max ^											
9 yo	42.48	0.34	576.56	41.63	43.34	41.97	0.39	576.56	40.99	42.96	0.13
12 yo	41.49	0.341	576.56	40.63	42.34	42.22	0.39	576.56	41.23	43.20	−0.21 ^#^

EMM = Estimate marginal mean; df = Degrees of freedom; CL = Confidence level; d = Effect size; ^#^ = Small practical significance; ^##^ = Medium practical significance; ^###^ = Strong practical significance; ^ = Not measured in 2010; yo = Years old.

**Table 5:** Interaction effect of age and gender for PF tests (*N* = 349).

Year Group	Boys (*n* = 165)					Girls (*n* = 184)					d
	EMM	SE	df	Lower CL	Upper CL	EMM	SE	df	Lower CL	Upper CL	
Sit-ups											
6 yo	4.07	0.40	873.67	3.01	5.13	4.13	0.40	873.67	3.09	5.18	−0.02
9 yo	19.03	0.40	873.67	17.98	20.09	16.22	0.40	873.67	15.17	17.26	0.65 ^##^
12 yo	12.95	0.40	873.67	11.89	14.01	10.36	0.40	873.67	9.32	11.41	0.60 ^##^
Push-ups											
6 yo	10.89	0.44	979.07	9.73	12.05	9.33	0.43	979.07	8.18	10.47	0.31 ^#^
9 yo	21.47	0.44	979.07	20.31	22.62	16.07	0.43	979.07	14.93	17.21	1.05 ^###^
12 yo	18.53	0.44	979.07	17.37	19.69	11.84	0.43	979.07	10.70	12.99	1.30 ^###^
VO_2_max ^											
9 yo	43.61	0.37	576.56	42.68	44.54	40.85	0.37	576.56	39.93	41.76	0.78 ^##^
12 yo	44.53	0.37	576.56	43.61	45.46	39.17	0.37	576.56	38.25	40.08	1.51 ^###^

EMM = Estimate marginal mean; df = Degrees of freedom; CL = Confidence level; d = Effect size; ^#^ = Small practical significance; ^##^ = Medium practical significance; ^###^ = Strong practical significance; ^ = Not measured in 2010; yo = Years old.

## Data Availability

If necessary, AP, the principal investigator of the research project, may be contacted to obtain the dataset.
