# Correction: Muller et al. Global Comparisons of Age, Gender and Socioeconomic Status Differences of Physical Fitness Health Risk in South African Primary School Children: Longitudinal Data from the NW-CHILD Study. *Int. J. Environ. Res. Public Health* 2024, *21*, 1554

**DOI:** 10.3390/ijerph23080988

**Published:** 2026-07-29

**Authors:** Xonné Muller, Anita E. Pienaar, Barry Gerber, Colin N. Moran, Naomi E. Brooks

**Affiliations:** 1 Physical Activity, Sport and Recreation Focus Area, Faculty of Health Science, North-West University, Potchefstroom 2520, South Africa; anita.pienaar@nwu.ac.za (A.E.P.); barry.gerber@nwu.ac.za (B.G.); 2 Department of Human Movement Sciences, University of Fort Hare, Alice 5700, South Africa; 3 Faculty of Health Sciences and Sport, University of Stirling, Scotland FK9 4LA, UK; colin.moran@stir.ac.uk (C.N.M.); n.e.brooks@stir.ac.uk (N.E.B.); 4 Department of Kinesiology, DePauw University, Greencastle, IN 46135-0037, USA

**Error in Table**

In the original publication [1], there was a mistake Table 1, regarding the maximum pacer laps for boys from low-SES at age 12. The correct Table 1 is as follows.

In the original publication [1], there was a mistake Table 2, regarding the some of the standing long jump distances and minimum value of wall-sit for low-SES boys. The correct Table 2 is as follows.

**Text Correction**

There was an error in the original publication [1] regarding the discussion of long jump values. A correction has been made to the Results Section, paragraph 4.

“Table 2 portrays similar descriptive statistics, also divided by gender in each SES group as in Table 1, but more specifically for each strength and aerobic sub-component. Increases in muscular strength in both SES groups were found for long jump and wall-sitting from ages 6–12. Boys and girls of a low SES achieved significantly (*p* < 0.01) shorter jumping distances at all ages, except for boys at the age of 12 (*p* = 0.45). At younger ages (6 and 9 years old), the high SES group held the wall-sit position for longer, although only significant at the age of 6 (*p* < 0.01). At the age of 12, non-significant (*p* = 0.98) differences were found between the low-SES (boys = 60 s; girls = 59 s) and high-SES (boys = 59 s; girls 57 s) groups in the wall-sit tests. Both genders show significant decreases in push-ups from the age of 9 (*p* < 0.01). In boys, both low (21 to 18 push-ups) and high (22 to 19 push-ups) SES groups decreased in push-up performance from the age of 9–12. Girls showed similar declining trends in performing modified push-ups (low SES = 15 to 10; high SES = 17 to 14) from the ages of 9–12. Similar trends are also observed for sit-ups where both boys (low SES = 17 to 11; high SES = 21 to 15) and girls (low SES = 15 to 8; high SES = 18 to 13) decreased significantly (*p* < 0.01) in repetition output from the age of 9. The low-SES (46 s) group of boys outperformed the high-SES (40 s) group for V-ups at the age of 6; however, this was non-significant. Thereafter, a plateau (similar or slight differences) is observed for V-ups at older ages in boys. In general, boys performed better than girls in aerobic endurance (VO_2_max) across both SES groups and time points (*p* < 0.01).”

There was an error in the original publication [1] regarding the discussion of long jump values. A correction has been made to the Discussion section, Comparing criteria reference standards (CRSs) and Interaction effects of age, gender and socioeconomic (SES) on muscular fitness (MF) and cardiorespiratory fitness (CRF), subsection Criteria reference standards (CRSs) and interaction effects of age and socioeconomic status (SES) for standing long jump (SLJ), Paragraph Number 1 and 3.

“SLJ is an indicator of general MF in youth and is one of the most utilized MF tests globally that assesses lower body strength and, more specifically, explosive strength [47]. Thomas et al. [48] reported age- and gender-specific PF percentile norms for children aged 6–18, including SLJ, based on data from seven European countries that revealed poorer performance than our participants. The 50th percentile values represent an average performance for boys of 124 cm at age 6, 133 cm at age 9 and 163.5 cm at age 12. Both low-SES (6 years = 91.4 cm; 9 years = 112.3 cm; 12 years = 137.7 cm) and high SES (6 = year = 106.7 cm; 9 years = 127.9 cm; 12 years = 140.1) groups from the current study performed below the reported 50th percentiles at all ages. The 50th percentiles for females, denoted as 115.5 cm at age 6, 122.5 cm at age 9 and 150.5 cm at age 12 [48], compared to 83.8 and 91.4 cm (6 years), 104.1 cm and 111.7 cm (9 years), 124.1 cm and 132.3 cm (12 years) of our group, also show that both low-SES and high-SES female groups performed below average at all three ages. Similar results were found when comparing the 50th percentile cut-offs reported by Tomkinson et al. [17] and Kolimechkov et al. [49]. Cut-points by Tomkinson [17] and Kolimechkov et al. [49] created comprehensive percentile scores for PF tests such as the SLJ, based on existing European data. In some cases, the low-SES groups significantly underperformed, falling below the 10th percentile, which is worrying. Compared to a South African study by Africa et al. [50] from the Western Cape Province, results indicated that boys (high SES = 120.6 cm, low SES = 113.0 cm compared to high SES = 106.7 cm, low SES = 91.4 cm) and girls (high SES = 108.0 cm, low SES = 105.2 cm compared to high SES = 91.4 cm, low SES = 83.8 cm) from this study achieved shorter jumping distances for SLJ at the age of 6. This comparison also yields differences in the South African child population based on the different regions in S.A. 

Cut-points by Castro-Piñero et al. [12] for SLJ are further linked to a healthy cardiovascular profile for children (6–9 years old) and adolescents (12–16 years old). These cut-points are 104.5 cm for boys and 81.5 cm for girls in the 6–9 age group [12]. In the current study, the average SLJ distances for children aged 6–9 were reported as ranging from 91.4 cm (low SES) to 127.9 cm (high SES) for boys and 83.1 cm (low SES) to 111.7 cm (high SES) for girls. The cut-points set by Castro-Piñero et al. [12] for the adolescent group are 140.5 cm for boys and 120.5 cm for girls. According to the current findings, boys and girls reached an average jumping distance of 137.7 cm (low SES) to 140.1 cm (high SES) and 124.1 cm (low SES) to 132.3 cm (high SES) at the age of 12, respectively. Only girls reached these cut points at ages 6–12. Based on these cut-points by Castro-Piñero et al. [12] that identify cardiovascular disease (CVD), South African boys are at risk and likely to develop CVD 2 years later. Our study found that low SES hinders SLJ distances, leading to health risks, especially in boys.”

These corrections were approved by the Academic Editor. The original publication has also been updated. The original publication has also been updated.

## Figures and Tables

**Table 1:** Descriptive characteristics of sociodemographic, anthropometric and physical fitness indicators at baseline and follow-up time point measurements (*N* = 349).

Variables	Boys (*n* = 165)						Girls (*n* = 184)					
	Low SES (*n* = 83)			High SES (*n* = 82)			Low SES (*n* = 118)			High SES (*n* = 66)		
	M ± SD	Min	Max	M ± SD	Min	Max	M ± SD	Min	Max	M ± SD	Min	Max
**Biographic and Anthropometric**												
Age (years)												
6 years old	6.76 ± 0.48	6.00	7.80	6.90 ± 0.55	6.10	7.80	6.70 ± 0.44	6.00	7.80	6.88 ± 0.54	6.10	7.70
9 years old	9.84 ± 0.38	8.99	10.67	10.04 ± 0.35	9.32	10.68	9.77 ± 0.35	9.00	10.68	10.01 ± 0.36	9.24	10.64
12 years old	12.84 ± 0.39	11.89	13.68	13.05 ± 0.35	12.34	13.68	12.78 ± 0.36	12.03	13.68	13.02 ± 0.36	12.26	13.65
Stature (cm)												
6 years old	116.99 ± 4.69	102.10	130.30	124.50 ± 5.89	112.20	141.70	117.06 ± 4.94	107.90	128.50	122.33 ± 6.01	109.70	134.70
9 years old	132.33 ± 4.99	117.00	147.10	139.77 ± 6.60	123.20	161.00	134.03 ± 6.00	121.40	151.00	138.91 ± 7.84	113.00	155.00
12 years old	147.78 ± 7.15	131.90	169.40	157.24 ± 8.34	139.40	178.80	151.98 ± 6.38	136.70	168.80	157.35 ± 7.37	143.60	185.90
Body mass (kg)												
6 years old	20.67 ± 2.52	16.40	30.50	26.76 ± 5.73	17.20	45.90	20.74 ± 3.06	15.08	29.70	24.18 ± 4.97	16.40	42.80
9 years old	28.58 ± 5.26	19.60	55.90	37.31 ± 9.86	22.60	65.60	30.13 ± 6.88	21.70	55.80	36.09 ± 8.78	22.20	64.70
12 years old	39.05 ± 9.31	23.10	84.80	52.35 ± 14.17	30.80	100.80	44.17 ± 10.05	28.80	78.50	52.19 ± 12.37	36.90	95.20
BMI (Kg/m^2^)												
6 years old	15.18 ± 1.46	12.90	22.90	16.50 ± 2.70	12.90	26.80	15.10 ± 5.00	12.70	20.70	16.10 ± 2.50	12.90	24.00
9 years old	16.27 ± 2.37	12.90	25.83	19.00 ± 3.90	13.30	30.80	16.70 ± 3.00	13.10	27.40	18.50 ± 3.80	14.20	29.70
12 years old	17.81 ± 3.23	14.00	32.00	21.10 ± 4.70	14.90	36.50	19.00 ± 3.60	13.90	33.70	21.10 ± 4.40	16.10	34.50
∑ skinfolds (mm)												
6 years old	12.40 ± 3.40	8.20	27.00	16.00 ± 8.00	8.00	47.00	15.00 ± 5.00	8.00	38.00	19.00 ± 7.00	9.00	40.00
9 years old	13.30 ± 7.30	7.00	57.50	22.00 ± 11.00	8.00	53.00	18.00 ± 8.00	8.00	46.00	25.00 ± 11.00	12.00	54.00
12 years old	15.10 ± 11.40	7.50	78.20	25.00 ± 15.00	9.00	76.00	22.00 ± 10.00	9.00	60.00	28.00 ± 14.00	14.00	75.00
Body fat (%)												
6 years old	14.60 ± 3.30	8.70	27.80	18.00 ± 6.00	10.00	39.00	14.00 ± 4.00	8.00	29.00	17.00 ± 6.00	8.00	32.00
9 years old	18.10 ± 6.70	5.00	38.40	23.00 ± 8.00	7.00	42.00	19.00 ± 8.00	5.00	44.00	24.00 ± 9.00	10.00	47.00
12 years old	17.30 ± 7.10	7.60	41.80	22.00 ± 9.00	5.00	45.00	23.00 ± 8.00	10.00	46.00	27.00 ± 8.00	11.00	48.00
**Physical Fitness**												
Strength (SS)												
6 years old	16.80 ± 2.90	9.00	23.00	16.80 ± 3.20	9.00	23.00	15.30 ± 3.40	6.00	22.00	16.30 ± 3.70	8.00	29.00
9 years old	17.70 ± 3.00	8.00	24.00	18.60 ± 3.00	10.00	27.00	15.90 ± 3.00	7.00	22.00	16.90 ± 3.60	10.00	25.00
12 years old	13.90 ± 2.20	7.00	18.00	14.50 ± 2.50	8.00	21.00	13.10 ± 2.10	7.00	19.00	14.60 ± 3.20	8.00	22.00
PACER (laps) ^												
9 years-old	27.00 ± 15.00	4.00	74.00	24.00 ± 13.00	5.00	65.00	17.00 ± 9.00	2.00	59.00	19.00 ± 9.00	7.00	45.00
12 years-old	37.00 ± 16.00	7.00	82.00	39.00 ± 19.00	8.00	83.00	20.00 ± 10.00	6.00	58.00	25.00 ± 17.00	5.00	80.00

BMI = Body mass index; M = Mean; Max = Maximum value; Min = Minimum value; SD = Standard deviation; SES = Socioeconomic status; ∑ skinfolds = Sum of sub-scapular + triceps skinfolds; SS = Scale score; ^ = Not measured in 2010.

**Table 2:** Descriptive characteristics of muscular strength and aerobic endurance sub-tests according to gender and SES over three time points (*N* = 349).

Variables	Boys (*n* = 165)						Girls (*n* = 184)					
	Low SES (*n* = 83)			High SES (*n* = 82)			Low SES (*n* = 118)			High SES (*n* = 66)		
	M ± SD	Min	Max	M ± SD	Min	Max	M ± SD	Min	Max	M ± SD	Min	Max
Long jump (cm)												
6 years old	91.40 ± 20.30	43.20	134.60	106.70 ± 15.20	139.70	168.66	83.08 ± 15.20	50.80	134.62	91.40 ± 15.20	50.80	134.00
9 years old	112.30 ± 21.49	75.01	172.72	127.86 ± 22.63	76.99	177.04	104.10 ± 17.80	58.40	149.90	111.70 ± 25.40	53.30	165.10
12 years old	137.74 ± 16.37	91.44	177.80	140.13 ± 23.52	79.50	195.58	124.06 ± 17.16	76.20	168.00	132.27 ± 20.43	69.12	197.12
Push-up (reps)												
6 years old	12.00 ± 6.00	0.00	26.00	10.00 ± 5.00	0.00	25.00	9.00 ± 6.00	0.00	20.00	10.00 ± 5.00	0.00	17.00
9 years old	21.00 ± 5.00	10.00	33.00	22.00 ± 5.00	11.00	40.00	15.00 ± 5.00	0.00	28.00	17.00 ± 6.00	5.00	30.00
12 years old	18.00 ± 7.00	2.00	35.00	19.00 ± 6.00	6.00	33.00	10.00 ± 5.00	0.00	26.00	14.00 ± 7.00	1.00	27.00
Sit-up (reps)												
6 years old	3.00 ± 3.00	0.00	11.00	5.00 ± 4.00	0.00	17.00	2.00 ± 3.00	0.00	12.00	6.00 ± 4.00	0.00	15.00
9 years old	17.00 ± 6.00	0.00	32.00	21.00 ± 6.00	2.00	50.00	15.00 ± 6.00	0.00	28.00	18.00 ± 5.00	0.00	32.00
12 years old	11.00 ± 4.00	0.00	19.00	15.00 ± 7.00	2.00	60.00	8.00 ± 5.00	0.00	21.00	13.00 ± 5.00	0.00	26.00
Wall-sit (s) *												
6 years old	49.00 ± 15.00	10.0	60.00	40.00 ± 20.00	6.00	60.00	50.00 ± 15.00	10.00	60.00	40.00 ± 19.00	8.00	60.00
9 years old	58.00 ± 7.00	23.00	60.00	54.00 ± 12.00	14.00	60.00	56.00 ± 10.00	20.00	60.00	54.00 ± 14.00	6.00	60.00
12 years old	60.00 ± 3.00	42.00	60.00	59.00 ± 4.00	34.00	60.00	59.00 ± 6.00	18.00	60.00	57.00 ± 9.00	10.00	60.00
V-up (s) *												
6 years old	46.00 ± 17.00	7.00	60.00	40.00 ± 19.00	0.00	60.00	41.00 ± 20.00	0.00	60.0	42.00 ± 18.00	0.00	60.00
9 years old	53.00 ± 12.00	14.00	60.00	56.00 ± 11.00	7.00	60.00	50.00 ± 17.00	0.00	60.0	52.00 ± 14.00	3.00	60.00
12 years old	53.00 ± 12.00	10.00	60.00	54.00 ± 11.00	10.00	60.00	50.00 ± 13.00	20.00	60.0	53.00 ± 14.00	5.00	60.00
VO_2_max (mL/kg/min) ^												
9 years old	44.20 ± 5.20	35.70	60.30	43.00 ± 4.40	35.80	57.30	40.80 ± 2.90	35.70	54.60	40.90 ± 3.20	36.30	50.90
12 years old	44.40 ± 5.50	33.30	59.70	44.60 ± 6.80	33.20	60.30	38.50 ± 3.60	33.20	51.30	39.80 ± 6.00	32.60	59.10

* 60 s was the maximum requirement to score full points for the wall-sit and V-up sub-tests. cm = Centimeters; M = Mean; Max = Maximum value; Min = Minimum value; reps = Repetitions; SD = Standard deviation; sec = Seconds; SES = Socioeconomic status; ^ = Not measured in 2010.
